# Compartment-specific investigations of antioxidants and hydrogen peroxide in leaves of *Arabidopsis thaliana* during dark-induced senescence

**DOI:** 10.1007/s11738-016-2150-6

**Published:** 2016-05-06

**Authors:** Nora Luschin-Ebengreuth, Bernd Zechmann

**Affiliations:** University of Teacher Education Styria, Hasnerplatz 12, 8010 Graz, Austria; Center for Microscopy and Imaging, Baylor University, One Bear Place #97046, Waco, TX 76798 USA

**Keywords:** *Arabidopsis*, Ascorbate, Glutathione, Hydrogen peroxide, Senescence, Transmission electron microscopy

## Abstract

The aim of this study was to gain insight into the compartment-specific roles of ascorbate and glutathione in leaf senescence in *Arabidopsis thaliana*. The subcellular distribution of ascorbate, glutathione, and hydrogen peroxide (H_2_O_2_) was analyzed by transmission electron microscopy and correlated with the activity of antioxidative enzymes in wildtype plants and the ascorbate- and glutathione-deficient mutants *vtc2*-*1* and *pad2*-*1*, respectively. Both mutants showed earlier and stronger senescence than the wildtype indicating the importance of a functioning ascorbate and glutathione cycle in the induction and regulation of senescence. Glutathione levels dropped drastically and up to 93 % in all cell compartments of wildtype plants and the *vtc2*-*1* mutant within the first day of dark-induced senescence while ascorbate contents remained unchanged until the very end. Glutathione contents in mitochondria of *pad2*-*1* mutants decreased more slowly over the first 7 days than compared to the other plants indicating an important role of glutathione in mitochondria in this mutant during senescence. The strongest decrease (84 %) of glutathione contents in wildtype plants at this time point was found in mitochondria indicating an important role of mitochondria for the induction of senescence and cell death events. Due to the general decrease of the antioxidative capacity, a strong accumulation of H_2_O_2_ was observed in cell walls, plastids, and the cytosol in all plants. Activities of glutathione reductase, dehydroascorbate reductase and catalase were strongly reduced while ascorbate peroxidase and monodehydroascorbate reductase were increased. The initial rapid drop of glutathione levels seemed to be the trigger for senescence, while ascorbate appeared to be the key factor in regulating senescence through controlling H_2_O_2_ levels by the oxidation of reduced ascorbate to monodehydroascorbate and the subsequent reduction to ascorbate by monodehydroascorbate reductase.

**Electronic supplementary material:**

The online version of this article (doi:10.1007/s11738-016-2150-6) contains supplementary material, which is available to authorized users.

## Introduction

Leaf senescence is a highly regulated process which follows a timetable and is determined by developmental and environmental factors (e.g. darkness, nutrient starvation, high or low sucrose or glucose contents, pathogen infection). This process is mediated by a genetic program and it is controlled by many different substances (reactive oxygen species, hormones, signal molecules, kinases and transcription factors) which guarantee that nutrients are remobilized from the senescing organs to support growth and development of younger ones (Thomas 2013). Leaf senescence is characterized by chlorophyll degradation, loss of proteins, and degeneration of nucleic acids which goes hand in hand with a controlled breakdown of cell and organelle structures. The earliest structural changes occur in plastids, such as degeneration of thylakoids, and increase in the number of plastoglobules (Austin et al. 2006; Ytterberg et al. 2006) while the nucleus and mitochondria remain functional until the very end to maintain gene expression and energy production (Guo and Gan 2005; Lim et al. 2007). Controlled vacuolar collapse, chromatin condensation and DNA-laddering, and disintegration of plasmamembrane occur in the later stages of senescence when programmed cell death (PCD) leads to the complete disintegration of the cell (Guo and Gan 2005; Lim et al. 2007).

Reactive oxygen species (ROS) play an important role during these processes as their accumulation during senescence leads to lipid peroxidation, provokes destructive protein modifications, induces mutagenic DNA strand breaks, and causes purine oxidations and protein-DNA-cross links. Furthermore, ROS are involved in senescence through the activation of gene expression, controlling hormone signal transduction and the modulation of specific genes essential for ROS-induced cell death (Mach and Greenberg 2004; Zimmermann and Zentgraf 2005; Zentgraf 2007; Mhamdi et al. 2010). The accumulation of ROS during senescence is accompanied by a loss of antioxidative capacity in plants and animals (Pastori and del Río 1997; Jiménez et al. 1998; Mach and Greenberg 2004; Panchuk et al. 2005; Palma et al. 2006; Zimmermann et al. 2006). Antioxidants regulate the content and subcellular location of ROS to ensure accurate execution of senescence signaling pathways and they must be precisely regulated during senescence to ensure the organized degradation of cell structures (Jones 2004; Lim et al. 2007; de Pinto et al. 2012). Ascorbate and glutathione are the most important antioxidants in plants and detoxify ROS either directly or through the ascorbate–glutathione cycle (Foyer and Noctor 2011; Zechmann 2014). The first step of this cycle uses ascorbate peroxidase (APX) to detoxify hydrogen peroxide (H_2_O_2_). The reduced form of ascorbate (Asc) is oxidized to monodehydroascorbate (MDHA) during this step. MDHA is either reduced by monodehydroascorbate reductase (MDHAR) to Asc or reacts to dehydroascorbate (DHA) which is then reduced by dehydroascorbate reductase (DHAR) to Asc. In this reaction the reduced form of glutathione (GSH) is oxidized to glutathione disulfide (GSSG) which is then further reduced by glutathione reductase (GR) to GSH. The electron acceptor NADP is regenerated during the reduction of MDHA and GSSG by the respective enzymes (Foyer and Noctor 2009). Ascorbate and glutathione can be found in most cell compartments (Zechmann and Müller 2010; Zechmann et al. 2011). Changes in total ascorbate and glutathione contents and adjustments of the ratio between their oxidized and reduced forms have been found to play an important regulatory role for the induction of PCD and during senescence (Jiménez et al. 1998; Noctor and Foyer 1998; Palma et al. 2006; Munné-Bosch et al. 2013). Decreased ascorbate levels for example appear to be a specific signal in TBY-2 cells undergoing PCD (de Pinto et al. 2012) and reduced ascorbate contents caused localized cell death and expression of PR-genes in *Arabidopsis vtc* mutants (Pavet et al. 2005). Glutathione levels and redox state in mitochondria and cytosol were shown to modify the cytochrome c-dependent activation of cell death (de Pinto et al. 2012). In animal cells glutathione depletion is a common feature of apoptotic cell death, and glutathione depletion was shown to regulate apoptosis triggered by a wide variety of stimuli (Franco and Cidlowski 2009). Promotion of cell death in animal cells was also closely correlated with glutathione depletion in mitochondria (Franco and Cidlowski 2009). A meta-analysis of several plants subjected to various stresses revealed that glutathione redox state is closely related to cell viability and that changes in GSSG/GSH ratios may be one of the signals triggering PCD (Kranner et al. 2006). Additionally, decreased glutathione levels were correlated to senescence of root nodules in legumes (Dalton et al. 1993; Evans et al. 1999; Puppo et al. 2005).

Even though it is commonly accepted that ascorbate and glutathione are involved in the development of senescence very little is known about their role during senescence at the subcellular level. A strong decrease in glutathione and ascorbate contents in mitochondria could be correlated with advanced senescence in pea leaves (Jiménez et al. 1998). A similar situation was found in tomato plants during the infection with *Botrytis cinerea* where the collapse of the antioxidative system in peroxisomes and mitochondria promoted leaf senescence (Kużniak and Skłodowska 2005a, b). Decreased levels of ascorbate and to some extent also glutathione were found in mitochondria, peroxisomes and plastids during leaf senescence in leaves of nodulated pea plants (Palma et al. 2006). Even though these investigations gave some insight into the importance of ascorbate and glutathione during senescence at the subcellular level it is still unclear how changes of ascorbate and glutathione contents in other cell compartments such as the nuclei, vacuoles, and the cytosol affect leaf senescence in plants.

Thus, the aim of this study was to investigate the role of subcellular ascorbate and glutathione levels during dark-induced senescence and correlate their compartment specific importance with the subcellular accumulation of H_2_O_2_, activities of antioxidative enzymes [GR, DHAR, APX, catalase (CAT)] and ultrastructural changes during senescence (e.g. plastid size, number and ultrastructure determined on cross sections) in *Arabidopsis thaliana*. The situation was monitored in wildtype plants and in mutants deficient in ascorbate (*vtc2*-*1* contains 60 % less ascorbate than the wildtype) and glutathione (*pad2*-*1* contains 80 % less glutathione than the wildtype) over a period of 10 days from the onset of senescence until the end when whole leaves disintegrated. The comparison between wildtype and the mutants was aimed to test the hypothesis that mutants with altered antioxidative capacity will show accelerated senescence when compared to the control.

## Materials and methods

### Plant material and growing conditions

*Arabidopsis thaliana* [L.] Heynh. Ecotype Columbia (Col-0) as well as the mutant lines *pad2*-*1* and *vtc2*-*1* were raised in growth cambers with 8:16 h, light:dark period at 22:18 °C. Six weeks after stratification fully developed leaves from the 3rd rosette of the plants were covered with aluminum foil. Dark-induced senescence is frequently used as a convenient method to synchronize the senescence process (Weaver et al. 1998; Weaver and Amasino 2001; Lin and Wu 2004; Buchanan-Wollaston et al. 2005; van der Graaff et al. 2006). Leaves were harvested 1, 2, 4, 7, and 10 days after the onset of dark-induced senescence. Control leaves remained uncovered.

### Sample preparation for transmission electron microscopy and immunogold labeling

Sample preparation for cytohistochemical detection of ascorbate and glutathione, and visualization of H_2_O_2_ by cerium chloride (CeCl_3_) was performed as described previously (Zechmann et al. 2008, 2011; Zechmann and Müller 2010; Heyneke et al. 2013). Briefly, sections of leaves were fixed in 2.5 % paraformaldehyde and 0.5 % glutaraldehyde for cytohistochemical investigations, rinsed in buffer, dehydrated in increasing concentrations of acetone, and gradually infiltrated with increasing concentrations of LR-White resin. Specimens were polymerized at 50 °C for 48 h under anaerobic conditions. Sections for H_2_O_2_ visualization were incubated in 5 mM CeCl_3_, fixed in 2.5 % glutaraldehyde, then rinsed in buffer, post-fixed in 1 % osmium tetroxide, dehydrated in increasing concentrations of acetone, and infiltrated with increasing concentrations of Agar 100 epoxy resin. Specimens were polymerized at 60 °C for 48 h. Sections for ultrastructural investigations were prepared as described above but without incubation in CeCl_3_. Ultra-thin sections (80 nm) were cut with a Reichert Ultracut S ultramicrotome (Leica Microsystems, Vienna, Austria).

Immunogold labeling of ascorbate and glutathione and evaluation of labeling through negative controls were done according to Zechmann et al. (2008, 2011). Sections were blocked with 2 % bovine serum albumine and then treated with the primary antibodies against ascorbate (anti-ascorbate IgG; Abcam plc, Cambridge, UK) diluted 1:300 and glutathione (EMD Millipore Corp., Billerica, MA, U.S.A.) diluted 1:50. After rinsing the sections in buffer, samples were incubated with secondary gold conjugated antibodies diluted 1:100 (for sections incubated with the ascorbate antibody) and 1:50 (for sections incubated with the glutathione antibody). Labeled grids were washed in distilled water, post stained with uranyl-acetate for 15 s, and investigated with a Philips CM10 transmission electron microscope (TEM). Gold particles were counted using the software package Cell F in the different cell compartments. A minimum of 20 (peroxisomes and vacuoles) to 60 (other cell structures) sectioned cell structures of at least 15 different cells were analyzed. The obtained data were statistically evaluated with SPSS Statistics (IBM Corp. New York, USA) by applying the Mann–Whitney *U* test. The specificity and accuracy of the immunogold localization methods have been demonstrated in detail in previous experiments (Zechmann et al. 2008, 2011; Zechmann and Müller 2010).

### Determination of plastid number and their fine structures

Changes in the number of plastids and their inner structures were evaluated according to Zechmann et al. (2003) and Heyneke et al. (2013). An Olympus AX70 light microscope (Olympus, Life and Material Science Europa GmbH, Hamburg, Germany) with a 40× objective lens was used to determine the number of sectioned plastids in the palisade cell layer and the spongy parenchyma by counting the plastids per cell on four semi-thin cross sections (3 µm) for each replicate sample. A minimum of 100 cells per leaf-type were examined to calculate the number of sectioned plastids in the cells. Ultra-thin sections were investigated with the TEM to determine changes in the ultrastructure of the plastids including the thylakoid-system, starch grains, and plastoglobules. These structures were then analyzed as digital images using the program Optimas 6.5.1 (BioScan Corp.). A minimum of 20 sectioned plastids from at least ten different cells from four different samples per leaf-type were examined. The obtained data were statistically evaluated with SPSS Statistics (IBM Corp. New York, USA) by applying the Mann–Whitney *U* test.

### Measurement of pigment content and activities of antioxidant enzymes

Content of chlorophyll a and b (Chl a, Chl b) and total carotenoids was analyzed in acetone extracts of frozen leaf material as described previously (Heyneke et al. 2013). Plant tissues were frozen and ground in liquid nitrogen. Chl and carotenoids were extracted with 100 % acetone in darkness at 4 °C for 20 min. The homogenate was centrifuged and pigment content was quantified spectrophotometrically by measuring the absorbance at 663, 645, and 470 nm on a UV-spectrophotometer (Hitachi U-3000). Pigment content was calculated as described previously (Heyneke et al. 2013). Enzyme activity was measured with a modified method according to Jiménez et al. (1997) and Koffler et al. (2014). Fresh leaf material was ground in liquid nitrogen and incubated for 30 min on ice with 1 % polyvinylpyrrolidone in extraction buffer (weight per volume) containing 100 mM NaH_2_PO_4_ (pH 7.5) and 1 mM EDTA. The homogenate was centrifuged at 4 °C and 20,000*g* for 10–30 min until a clear supernatant was obtained. All reactions were carried out in a total volume of 500 µl and measured in UV-permeable plastic cuvettes (Brand, Wertheim, Germany) at 25 °C against reagent blank on a UV–VIS spectrophotometer (Hitachi U-3000). APX activity was measured as the decrease in absorbance at 290 nm due to ascorbate oxidation (*ε*_290_ = 2.8 mM^−1^ cm^−1^) in 100 mM NaH_2_PO_4_ (pH 7.5) buffer containing 1 mM EDTA, 0.2 mM H_2_O_2_, 0.5 mM Asc and enzyme extract. For CAT activity measurement protein extract was added to a solution of 100 mM NaH_2_PO_4_ and 1 mM EDTA at pH 7.5. The reaction was started by adding H_2_O_2_ to a final concentration of 40 mM. The decrease of H_2_O_2_ was observed at 240 nm (*ε*_240_ = 0.04 mM^−1^ cm^−1^). DHAR was assayed in 100 mM NaH_2_PO_4_ (pH 7.0), 1 mM EDTA buffer containing 0.2 mM DHA, 2.5 mM reduced glutathione and plant extract by the increase in absorbance at 265 nm (*ε*_265_ = 14 mM^−1^ cm^−1^). GR activity was assayed by following the oxidation of NADPH at 340 nm (*ε*_340_ = 6.22 mM^−1^ cm^−1^). Reaction mixture contained 100 mM NaH_2_PO_4_ (pH 7.5), 1 mM EDTA buffer, 0.1 mM NADPH, 0.5 mM oxidized glutathione and enzyme extract. MDHAR activity was measured by the decrease of NADH at 340 nm (*ε*_340_ = 6.22 mM^−1^ cm^−1^) in a mixture of enzyme extract with 100 mM NaH_2_PO_4_ (pH 7.5) and 1 mM EDTA containing 3 mM ascorbate, 0.25 mM NADH, and 0.1 U ascorbate oxidase. The production of MDHA was monitored at 260 nm. The obtained data were statistically evaluated with SPSS Statistics (IBM Corp. New York, USA) by applying the Mann–Whitney *U* test.

## Results

### Visible symptoms and pigment contents

**Fig. 1:**
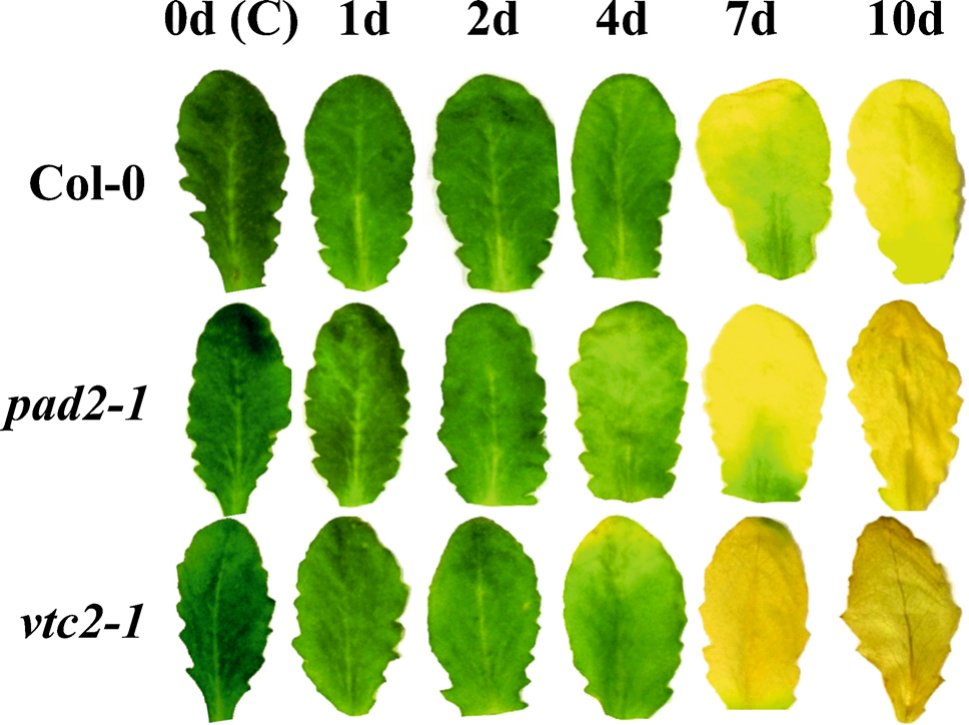
Visible changes of leaves during dark-induced senescence. Representative images of leaves from *Arabidopsis thaliana* Col-0 and the mutants *pad2*-*1* and *vtc2*-*1* throughout the dark-treatment experiment. First symptoms of senescence such as* yellowing* of the leaves could be observed in both mutants 4 days after dark treatment while wildtype plants did not show such symptoms at this time point. Leaves of both mutants showed more advanced yellowing of the leaves at later time points (7 and 10 days) when compared to the wildtype

**Fig. 2:**
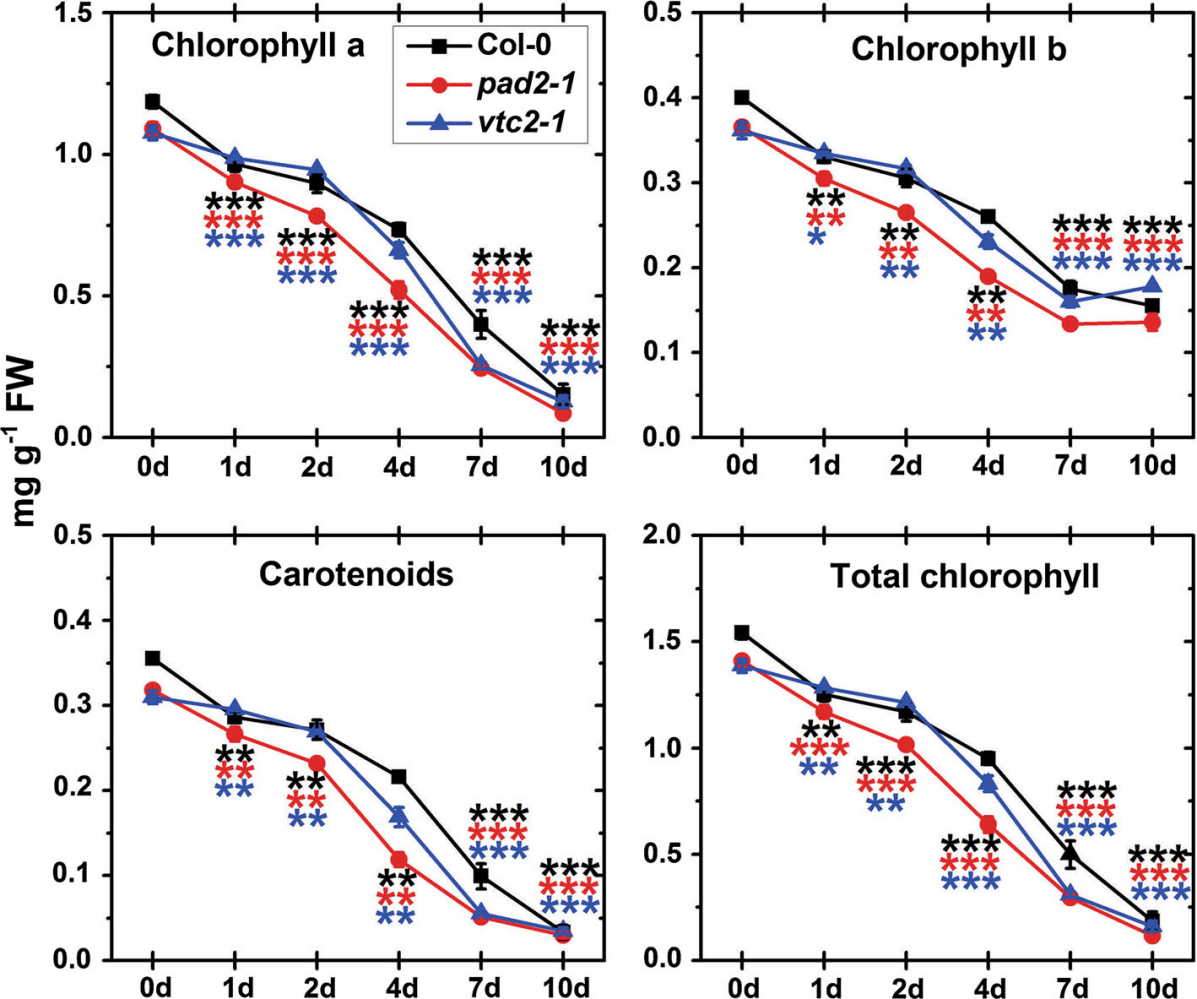
Pigment contents in leaves during dark-induced senescence. *Graphs* show contents of chlorophyll a, chlorophyll b, carotenoids, and total chlorophyll in leaves of *Arabidopsis* wildtype plants, and the *pad2*-*1* and *vtc2*-*1* mutants under control conditions (0 days) and at several time points throughout dark-induced senescence. Data are means with standard errors. Significant differences were calculated between samples taken at the beginning of the experiment (0 days) and later time points within wildtype plants, *pad2*-*1* and *vtc2*-*1* using the Mann–Whitney *U* test; *, ** and *** significance at a level of confidence of *p* < 0.05, *p* < 0.01 and *p* < 0.001, respectively. *ns* not significant different; *n* > 12. Significant differences between plants at one specific time point are shown in Tab. A1

First signs of senescence (yellowing of the leaves) could be observed in both mutants 4 days after dark treatment when wildtype plants did not show such symptoms yet (Fig. 1). Both mutants showed more advanced yellowing of the leaves after 7 days of dark treatment when compared to wildtype plants (Fig. 1). Leaves of all plants were completely yellow 10 days after dark treatment (Fig. 1). Pigment contents dropped in all plants starting 1 day after dark treatment (Fig. 2). Significantly less chlorophyll a, b, carotenoids, and total chlorophyll were found in Col-0 (between 18 and 19 %), *pad2*-*1* (16–17 %) and *vtc2*-*1* (5–8 %) at this time point. At the end of the experiment all plants contained about 90 % less chlorophyll and between 50 % (*pad2*-*1*) to 60 % (Col-0 and *vtc2*-*1*) less carotenoid when compared to the beginning of the dark treatment (Fig. 2).

### Light microscopical investigations

**Fig. 3:**
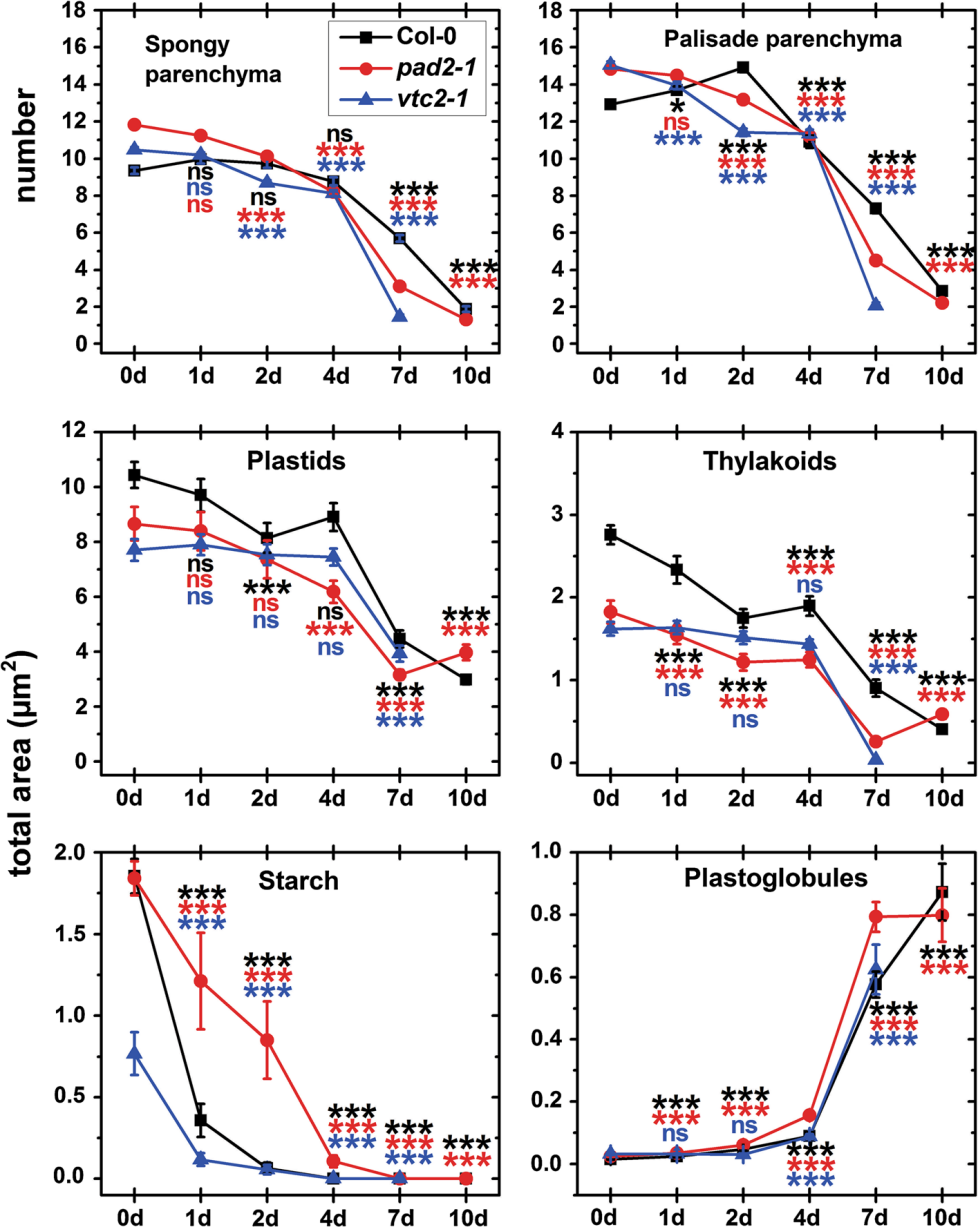
Changes in plastid number and structures during dark-induced senescence. *Graphs* show changes in the number of plastids in spongy and palisade parenchyma cells and changes in the area (µm^2^) of plastid fine structure on semi- and ultra-thin sections, respectively, throughout dark-induced senescence. The number of plastids was determined by image analysis on semi-thin cross sections (3 µm) of leaves with a light microscope by counting the plastids per sectioned cell in four replicate samples. Changes of the area (µm^2^) of plastid fine structures were determined by TEM and image analysis on TEM-micrographs of ultra-thin sections (80 nm) of plastids containing thylakoid-system, starch grains, and plastoglobules. Data are means with standard errors. Significant differences were calculated between samples taken at the beginning of the experiment (0 days) and later time points within wildtype plants, *pad2*-*1* and *vtc2*-*1* using the Mann–Whitney *U* test; *, ** and *** significance at a level of confidence of *p* < 0.05, *p* < 0.01 and *p* < 0.001, respectively. *ns* not significantly different; *n* > 100 cells or plastids. Significant differences between plants at one specific time point are shown in Tab. A1

Light-microscopic investigation of leaf cross sections revealed strong alterations especially of plastid number and size during dark-induced senescence. At the beginning of dark treatment leaves from all plants showed large cells with a central vacuole (Figs. 3, A1). Wildtype plants contained about 13 and 9 plastids per cell section in palisade and spongy parenchyma, respectively, and contained the largest plastids with an average size on semi-thin cross sections of about 10.4 μm^2^ (Fig. 3; Tab. A1). A higher number of plastids per cell section was found in *pad2*-*1* and *vtc2*-*1* mutants which contained about 15 plastids per cell section in palisade parenchyma and 12 (*pad2*-*1*) and 11 (*vtc2*-*1*) plastids in the spongy parenchyma with an average size on semi-thin cross sections of about 8 μm^2^ (Fig. 3; Tab. A1). Mutants showed significantly decreased amounts of plastids per cell section much earlier than wildtype plants. One day after dark treatment *vtc2*-*1* mutants showed 7 and 17 % less plastids per cell section in palisade and spongy parenchyma cells (Fig. 3). Significantly decreased amounts of plastids were observed 2 days after dark treatment in *pad2*-*1* mutants when 6 and 14 % less plastids were found in cell sections of palisade and parenchyma cells. Wildtype plants showed significantly decreased amounts of plastids per cell section 4 days after dark treatment when 15 and 7 % less plastids were found in palisade and spongy parenchyma cells (Fig. 3). At this time point plastid number was decreased in the mutants between 23 and 30 %. At the end of the experiment (10 days after dark treatment) a strong decrease in plastid number could be observed of up to 80 % in wildtype and 89 % in *pad2*-*1* mutants (Fig. 3). Ten days after dark treatment the ultrastructure of *vtc2*-*1* mutants had fully disintegrated rendering the quantification of the number of chloroplasts impossible (Fig. A1).

### Electron microscopical studies

A general decrease in the size of plastids on ultra-thin cross sections was observed after dark treatment in all plants. At the beginning of dark treatment plastids on ultra-thin cross sections had an average area of 10.44 µm^2^ in Col-0, 8.66 µm^2^ in *pad2*-*1* and 7.71 µm^2^ in *vtc2*-*1*. Interestingly, wildtype plants showed significant decrease (22 %) in plastid size on ultra-thin cross sections 2 days after dark treatment, whereas *pad2*-*1* and *vtc2*-*1* showed a decrease (28 and 49 %) 4 and 7 days after dark treatment (Fig. 3). At the end of the experiment (10 days after dark treatment) plants showed significantly smaller plastids on ultra-thin cross sections (71, 54 and 100 % for Col-0, *pad2*-*1*). Ten days after dark treatment the ultrastructure of *vtc2*-*1* mutants had fully disintegrated rendering the determination of the size of chloroplasts impossible (Fig. A2). A strong change in plastid fine structure on ultra-thin cross sections was found throughout the experiment. The most striking effect was the decrease in the size of starch grains on ultra-thin cross sections of 81, 34, and 84 % in Col-0, *pad2*-*1,* and *vtc2*-*1*, respectively, which started 1 day after dark treatment (Fig. 3). The size of starch grains found on ultra-thin cross sections of plastids was reduced to zero level in all samples 4 days after dark treatment (Figs. 3, A2). A strong decrease in thylakoid area was observed on ultra-thin cross sections in all samples which started in Col-0 1 day (16 %), in *pad2*-*1* (32 %) 2 days and in *vtc2*-*1* (−98 %) 7 days after dark treatment, resulting in 85 % (Col-0) and 68 % (*pad2*-*1*) less thylakoid area 10 days after dark-induced senescence (Fig. 3). Ten days after dark treatment, the ultrastructure of *vtc2*-*1* mutants had fully disintegrated rendering investigations of thylakoid impossible (Fig. A2).Throughout this development thylakoids were found to accumulate towards the plastid envelope leaving a large open area of stroma in the center. The decrease in thylakoid area on ultra-thin cross sections of plastids correlated with a massive increase in the area and size of plastoglobules on ultra-thin cross sections of plastids (Figs. 3, A2). At the end of the experiment plastid ultrastructure resulted in the formation of vesicles, large plastoglobules, remnants of thylakoid membranes, and dissolved stroma (Fig. A2). On many occasions the plastid envelope was ruptured and the plastid content was released into the vacuole (Fig. A2).

### Subcellular distribution of ascorbate during senescence

**Fig. 4:**
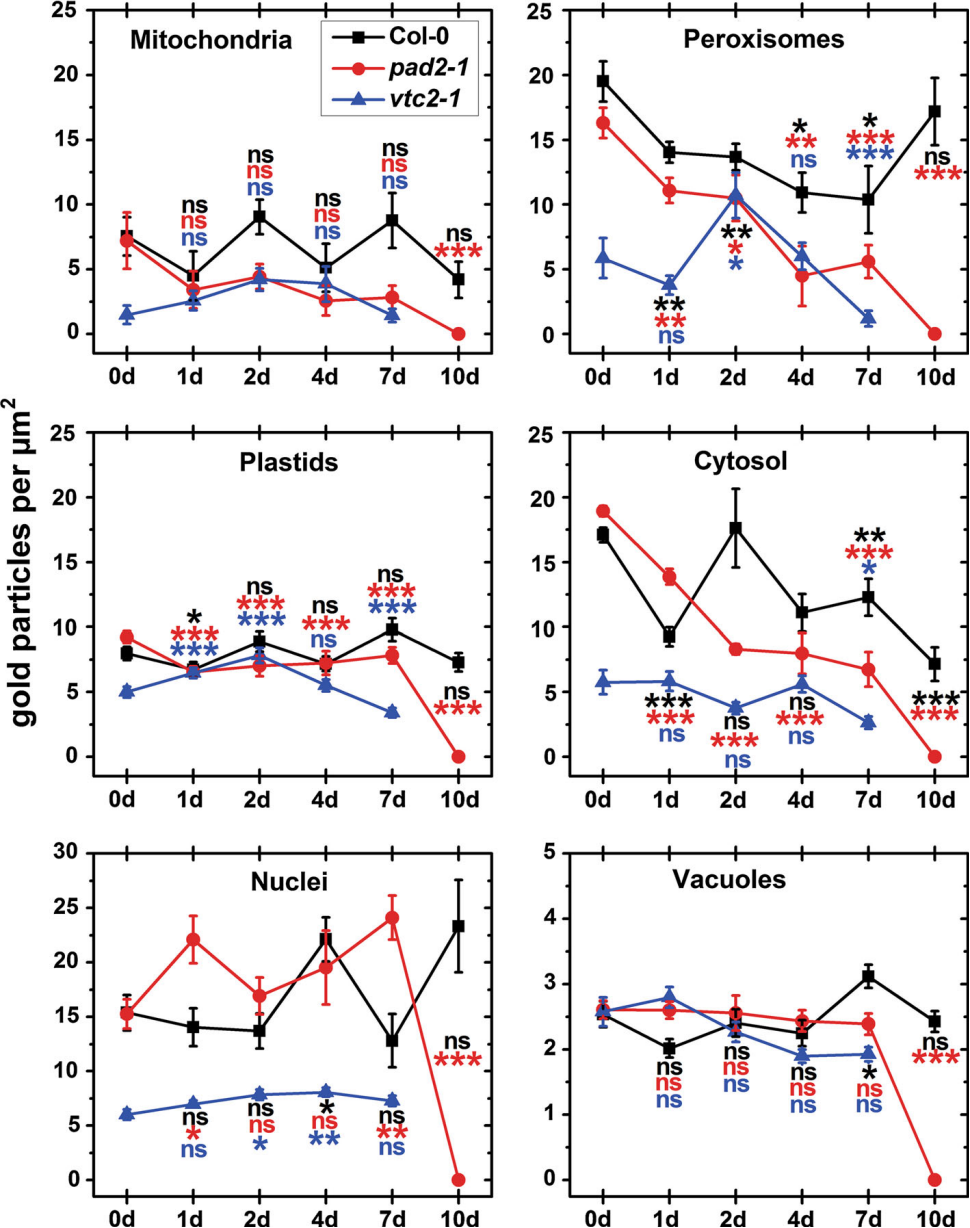
Compartment-specific ascorbate labeling density during dark-induced senescence. *Graphs* show the numbers of gold particles bound to ascorbate per μm^2^ in mesophyll cells of *Arabidopsis thaliana* Col-0 plants and the mutants *pad2*-*1* and *vtc2*-*1* at the beginning of the experiment (0 days) and throughout dark-induced senescence. Data show means with standard errors. Significant differences were calculated between samples taken at the beginning of the experiment (0 days) and later time points within wildtype plants, *pad2*-*1* and *vtc2*-*1* using the Mann–Whitney *U* test; *, ** and *** significance at a level of confidence of *p* < 0.05, *p* < 0.01 and *p* < 0.001, respectively. *ns* not significantly different. *n* > 20 for peroxisomes and vacuoles and *n* > 60 for other cell structures. Significant differences between plants at one specific time point are shown in Tab. A2

At the beginning of the experiment *Arabidopsis* Col-0 leaves showed highest density of gold particles per µm^2^ bound to ascorbate in peroxisomes (19.5), the cytosol (17.1) and the nuclei (15.4), respectively (Figs. 4, A3). In mitochondria, plastids and the vacuole 7.5, 8, and 2.5 gold particles bound to ascorbate per µm^2^ could be detected at this time point, respectively (Figs. 4, A3). Ascorbate contents varied strongly in the different cell compartments throughout the experiment. While mitochondria did not show significant changes throughout the experiment, plastids, nuclei, and vacuoles showed significant changes at 1 day (15 %), 4 days (44 %), and 7 days (22 %), respectively. The cytosol showed a strong decrease of ascorbate contents of up to 58 % in wildtype plants at the end of the experiment (7 and 10 days) while peroxisomes showed decreased levels of up to 46 % throughout the experiment (Figs. 4, A3).

Ascorbate-specific labeling in *pad2*-*1* mutants at the beginning of the experiment was similar to what was observed in wildtype plants (Tab. A2). Highest ascorbate labeling at this time was found in the cytosol, peroxisomes, and nuclei (18.9, 16.3, and 15.3 gold particles per µm^2^, respectively), followed by plastids, mitochondria, and the vacuole (9.22, 7.21, and 2.61 gold particles per µm^2^, respectively) (Figs. 4, A3). Dark-induced senescence resulted in an immediate (1 day) significant decrease of ascorbate contents in plastids (29 %), the cytosol (27 %), and peroxisomes (32 %) in this mutant while all other cell compartments contained high ascorbate contents until the very end of the experiment. At the end of the experiment (10 days) ascorbate contents were decreased to zero levels in all cell compartments (Figs. 4, A3).

As expected, leaves of *vtc2*-*1* mutants at the beginning of the experiment showed much lower ascorbate-specific labeling than wildtype plants or the *pad2*-*1* mutants (Tab. A2). These results were similar to recent reports which showed that the *vtc2*-*1* mutant contained between 50 and 60 % less ascorbate than wildtype plants (Zechmann et al. 2011). At the beginning of the experiment highest ascorbate-specific labeling was found in the nuclei, peroxisomes, and the cytosol (about 6 gold particles per µm^2^). Lower contents were found in plastids, vacuoles, and mitochondria (5, 2.6, and 1.5 gold particles per µm^2^) at this time point (Figs. 4, A3). Ascorbate contents remained unaffected by dark-induced senescence (mitochondria and vacuoles) for the first 7 days or were even slightly increased (plastids, nuclei, peroxisomes) for the first 2 days. Decreased amounts of ascorbate-specific labeling could be observed after 7 days of dark-induced senescence in plastids (32 %), peroxisomes (80 %), and the cytosol (54 %). Due to lack of ultrastructural preservation, ascorbate-specific labeling could not be evaluated anymore in *vtc2*-*1* at the end of the experiments (Figs. 4, A3).

### Subcellular distribution of glutathione during senescence

**Fig. 5:**
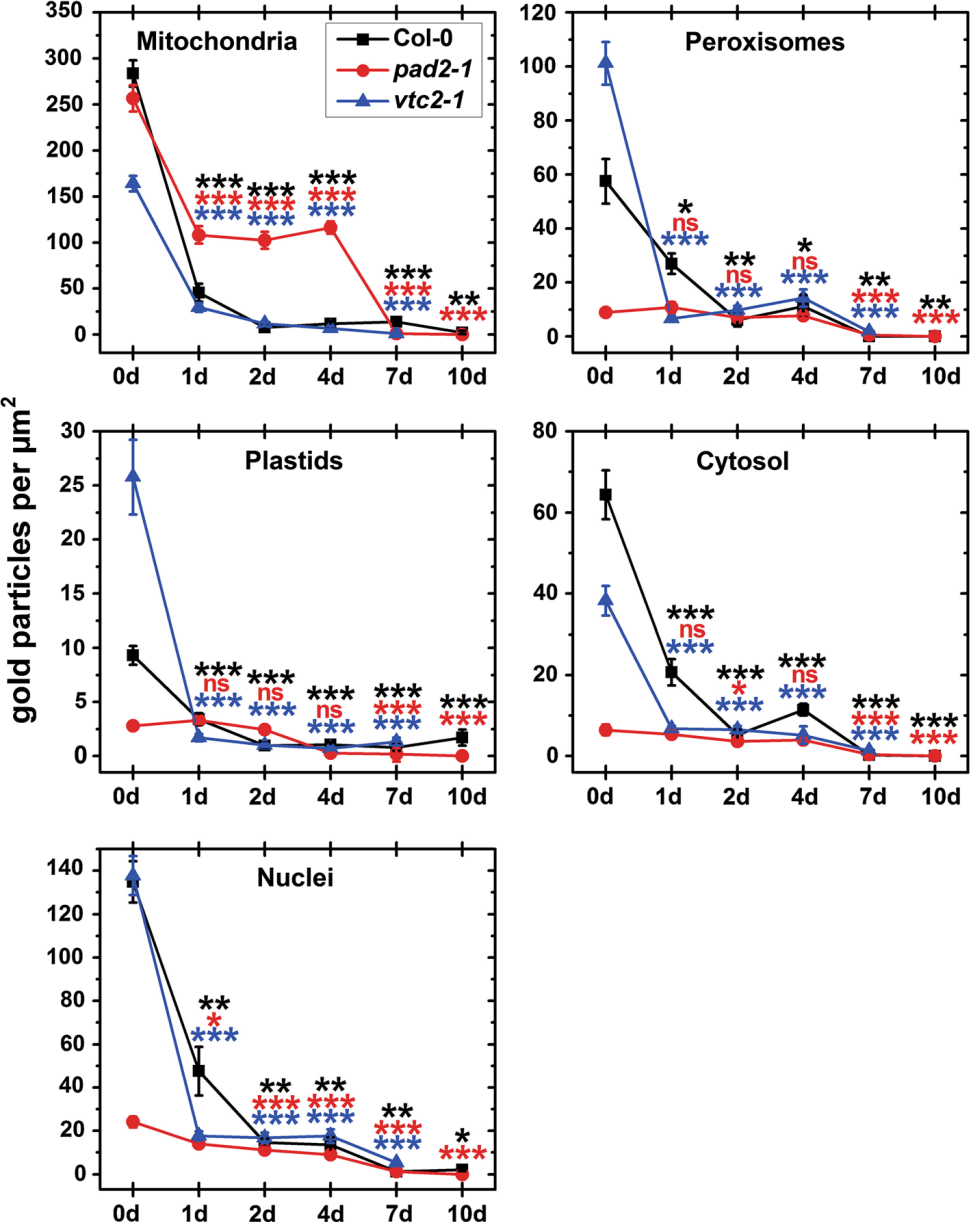
Compartment-specific glutathione labeling density during dark-induced senescence. *Graphs* show the numbers of gold particles bound to glutathione per μm^2^ in mesophyll cells of *Arabidopsis thaliana* Col-0 plants and the mutants *pad2*-*1* and *vtc2*-*1* at the beginning of the experiment (0 days) and throughout dark-induced senescence. Data show means with standard errors. Significant differences were calculated between samples taken at the beginning of the experiment (0 days) and later time points within wildtype plants, *pad2*-*1* and *vtc2*-*1* using the Mann–Whitney *U* test; *, ** and *** significance at a level of confidence of *p* < 0.05, *p* < 0.01, and *p* < 0.001, respectively. *ns* not significantly different. *n* > 20 for peroxisomes and vacuoles and *n* > 60 for other cell structures. Significant differences between plants at one specific time point are shown in Tab. A2

At the beginning of the experiment *Arabidopsis* Col-0 leaves showed highest density of gold particles per µm^2^ bound to glutathione in mitochondria (284) and nuclei (135) followed by the cytosol, peroxisomes, and plastids (63.34, 57.56, and 9.32 gold particles per µm^2^, respectively) (Figs. 5, A4). Gold particle density was strongly reduced within 1 day of dark-induced senescence in all cell compartments between 53 % in peroxisomes and 84 % in mitochondria. On the second day of dark induced senescence levels were decreased even further of about 97 % in mitochondria, and about 90 % in all other cell compartments. An almost 100 % decrease of glutathione contents could be observed in all cell compartments 7 days after dark-induced senescence (Figs. 5, A4).

At the beginning of the experiment the glutathione-deficient *pad2*-*1* mutant showed reduced glutathione-specific labeling in all cell compartments except mitochondria where levels similar to the wildtype were found (Tab. A2). These results are similar to what has been reported recently for this mutant which contained wildtype glutathione levels in mitochondria, whereas all other cell compartments contained up to 90 % less glutathione (Zechmann et al. 2008). At the beginning of the experiment 2.8, 24, 6.5, and 9 gold particles per µm^2^ were found in plastids, nuclei, the cytosol, and peroxisomes of *pad2*-*1* plants, respectively (Figs. 5, A4). Only in mitochondria of *pad2*-*1* glutathione-specific labeling was similar (257 gold particles per µm^2^) to values found in Col-0 plants. During dark-induced senescence a strong drop in glutathione contents could be observed within the first day in mitochondria (58 %) and nuclei (42 %) while glutathione contents in the other cell compartments remained close to control levels (Figs. 5, A4). Whereas glutathione contents in plastids, nuclei, peroxisomes, and the cytosol were similar to levels found in the wildtype and *vtc2*-*1* mutants until 7 days after dark-induced senescence glutathione levels in mitochondria of the *pad2*-*1* mutants remained much higher (up to 1236 % at 2 days when compared to Col-0). 7 days after dark-induced senescence glutathione contents were decreased in all cell compartments of the *pad2*-*1* mutant between 95 % (mitochondria) and 99 % (plastids and nuclei). At the end of the experiment, all cell compartments showed a 100 % decrease in glutathione contents when compared to the beginning of the experiment (Figs. 5, A4).

Leaves of the *vtc2*-*1* mutant plants contained highest glutathione-specific labeling at the beginning of the experiment in mitochondria, nuclei, and peroxisomes (164.19, 137.71 and 101.19 gold particles per µm^2^, respectively) followed by the cytosol and plastids (38.24 and 25.77 gold particles per µm^2^, respectively) (Figs. 5, A4). Glutathione-specific labeling strongly decreased within the first day of dark-induced senescence in all cell compartments between 82 % (mitochondria and the cytosol) and 93 % (plastids and peroxisomes). A further decrease of glutathione contents to about 95–100 % in all cell compartments could be observed 7 days after dark-induced senescence. Due to lack of ultrastructural preservation glutathione-specific labeling could not be evaluated anymore in *vtc2*-*1* at the end of the experiments (Figs. 5, A4).

### Activities of antioxidant enzymes

**Fig. 6:**
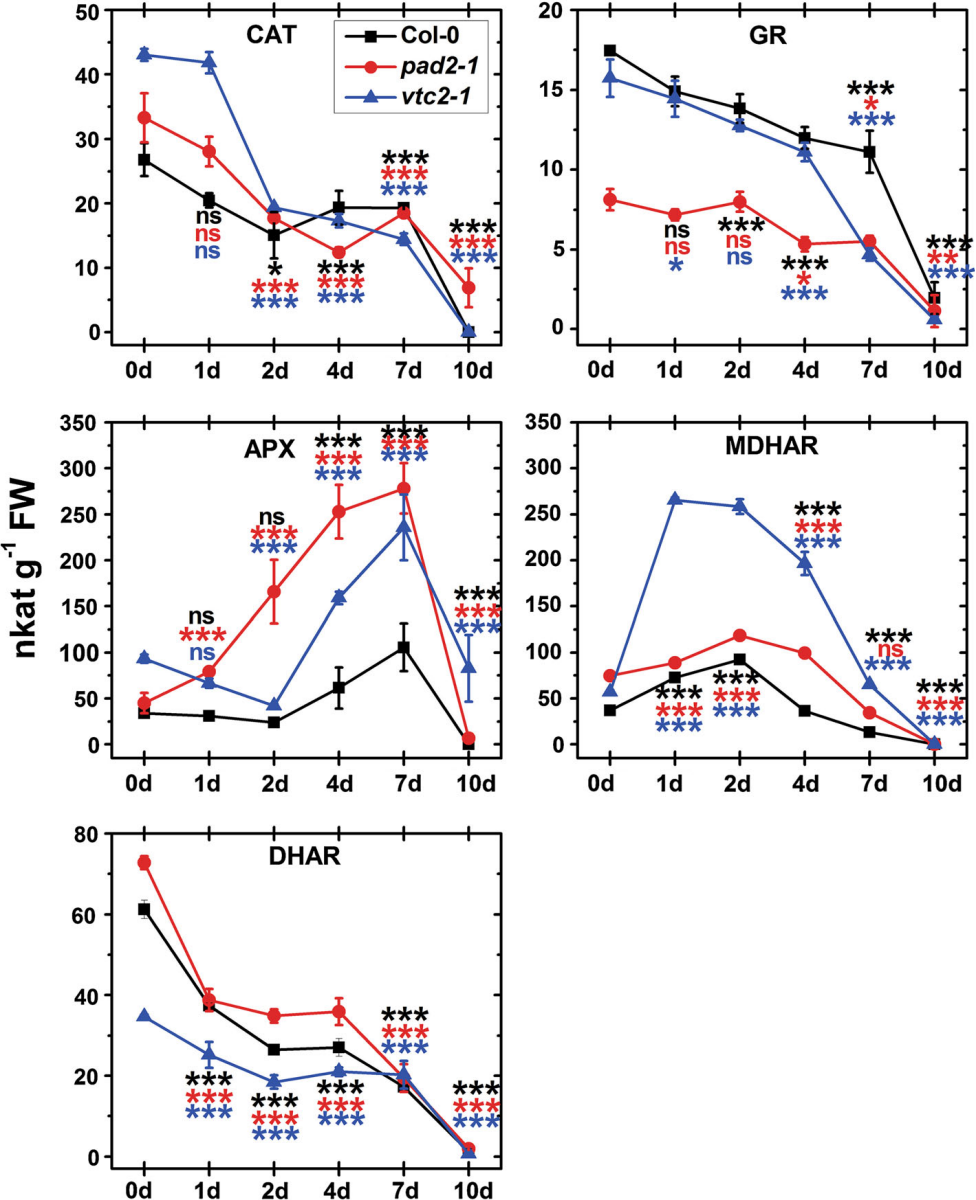
Activity of antioxidative enzymes during dark-induced senescence. Graphs demonstrate the activities of CAT, GR, APX, MDHAR, and DHAR in leaves of *Arabidopsis thaliana* Col-0 plants and the mutants *pad2*-*1* and *vtc2*-*1* at the beginning of the experiment (0 days) and throughout dark-induced senescence. Data show means with standard errors. Significant differences were calculated between samples taken at the beginning of the experiment (0 days) and later time points within wildtype plants, *pad2*-*1* and *vtc2*-*1* using the Mann–Whitney *U* test; *, ** and *** significance at a level of confidence of *p* < 0.05, *p* < 0.01 and *p* < 0.001, respectively. *ns* not significantly different; *n* > 12. Significant differences between plants at one specific time point are shown in Tab. A3

Activities of CAT, APX, GR, MDHAR, and DHAR were measured in whole leaf extracts. Under control conditions the ascorbate-deficient *vtc2*-*1* mutant showed highest CAT activity with 43 nkat g^−1^ FW, followed by *pad2*-*1* and Col-0 with 33 and 27 nkat g^−1^ FW, respectively (Fig. 6; Tab. A3). CAT activity significantly decreased in all *Arabidopsis* lines after 2 days of dark incubation to 15, 18, and 19 nkat g^−1^ FW in Col-0, *pad2*-*1,* and *vtc2*-*1*, respectively. Lower CAT activity was maintained during dark-induced senescence in all investigated plants. At the end of the experiment (10 days) CAT activity was at 7 nkat g^−1^ FW in *pad2*-*1* mutants while no activity could be detected in wildtype plants and the *vtc2*-*1* mutant (Fig. 6).

At the beginning of the experiment Col-0 showed highest GR activities of 17 nkat g^−1^ FW followed by *vtc2*-*1* with 16 nkat g^−1^ FW, and the glutathione-deficient mutant *pad2*-*1* with 8 nkat g^−1^ FW (Fig. 6; Tab. A3). Major changes in GR activities were found 4 and 7 days after dark-induced senescence when GR activity dropped to 11 nkat g^−1^ FW in wildtype plants and 5 nkat g^−1^ FW in both mutants. At the end of the experiment (10 days) GR activity was reduced to 3, 1, and 0 nkat g^−1^ FW in wildtype plants, *pad2*-*1,* and *vtc2*-*1* mutants, respectively (Fig. 6).

At the beginning of the experiment APX showed a significantly higher activity in *vtc2*-*1* leaves (93 nkat g^−1^ FW) when compared to *pad2*-*1* and wildtype (45 and 34 nkat g^−1^ FW, respectively) (Fig. 6; Tab. A3). In wildtype plants and the *vtc2*-*1* mutants a significant increase of APX activity up to 121 and 236 nkat g^−1^ FW, respectively, could be observed between 4 and 7 days after dark-induced senescence. The glutathione-deficient *pad2*-*1* mutant showed even higher APX activity during dark-induced senescence. A significant increase of APX activity in this mutant was observed after 1 day of dark treatment (79 nkat g^−1^ FW) and increased further to 253 and 278 nkat g^−1^ FW after 4 and 7 days of dark incubation, respectively (Fig. 6). At the end of the experiment (10 days) CAT activity was at 24 and 83 nkat g^−1^ FW in *pad2*-*1 and vtc2*-*1*, respectively, but could not be measured anymore in wildtype plants (Fig. 6).

At the beginning of the experiment MDHAR activity was found at 37, 75, and 57 nkat g^−1^ FW in wildtype plants, *pad2*-*1,* and *vtc2*-*1* mutants, respectively (Fig. 6; Tab. A3). A strong increase was found within 1 day of dark-induced senescence to 73, 88, and 266 nkat g^−1^ FW wildtype plants, *pad2*-*1,* and *vtc2*-*1* mutants, respectively. MDHAR activity increased (to 92 and 119 nkat g^−1^ FW in wildtype and *pad2*-*1* mutant) or remained at high levels until 4 days into dark-induced senescence. At the end of the experiment MDHAR activity was decreased to 13 and 34 nkat g^−1^ FW in wildtype plants and the *pad2*-*1* mutant and still slightly higher (65 nkat g^−1^ FW) in the *vtc2*-*1* mutant when compared to the situation at the beginning of the experiment (Fig. 6).

At the beginning of the experiment, DHAR activity was significantly higher in Col-0 and *pad2*-*1* with 61 and 73 nkat g^−1^ FW, respectively, when compared to *vtc2*-*1* with 35 nkat g^−1^ FW (Fig. 6; Tab. A3). DHAR activity dropped in all plants within the 1 day after dark-induced senescence to about 38 nkat g^−1^ FW in wildtype plants and the *pad2*-*1* mutant and 25 nkat g^−1^ FW in the *vtc2*-*1* mutant. DHAR activity dropped further to 17 nkat g^−1^ FW in wildtype plants and 20 nkat g^−1^ FW in both mutants 7 days after dark-induced senescence (Fig. 6). At the end of the experiment DHAR activity was not detected anymore.

### H_2_O_2_ accumulation during dark-induced senescence

**Fig. 7:**
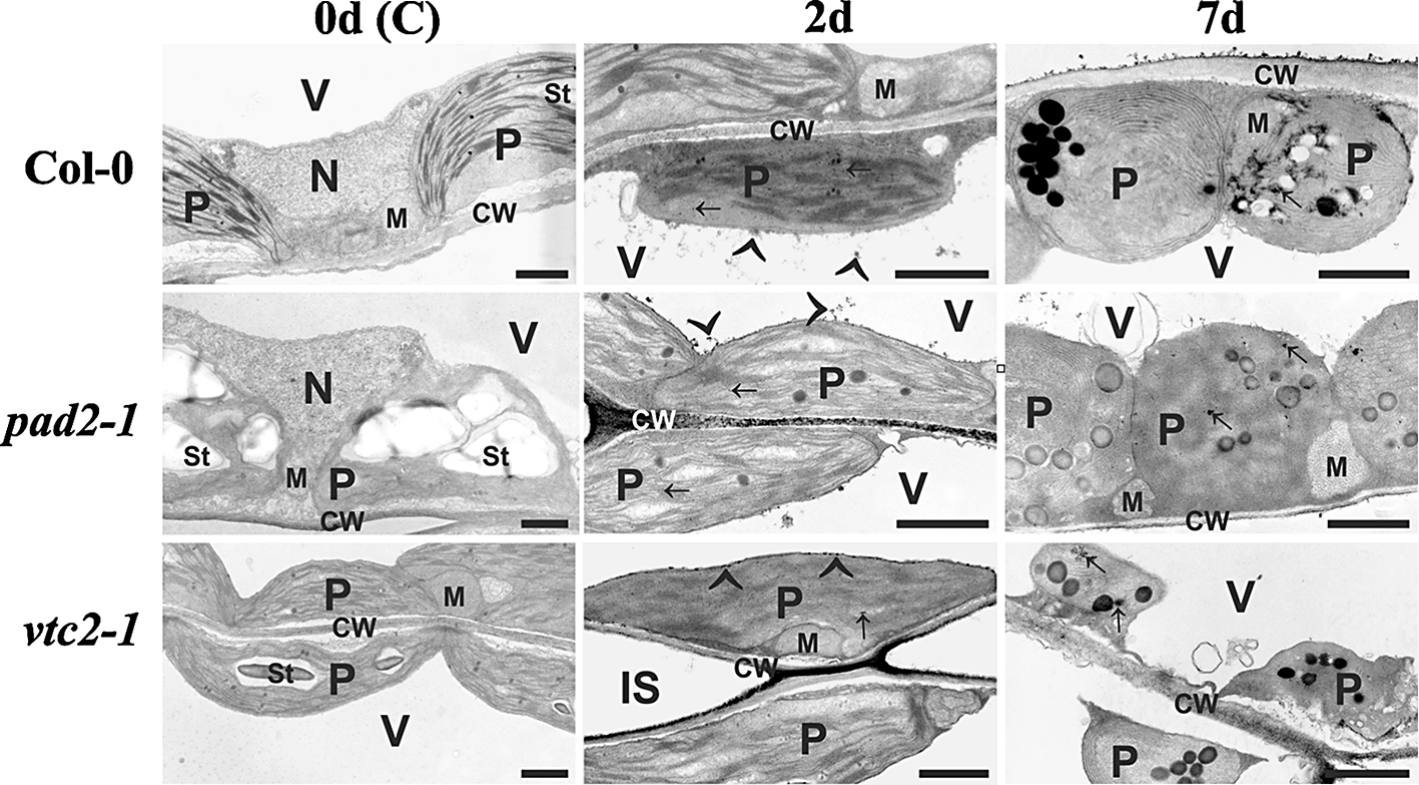
Subcellular distribution of H_2_O_2_ during dark-induced senescence. TEM-micrographs demonstrating the subcellular distribution of H_2_O_2_ visualized by CeCl_3_-staining in leaves of *Arabidopsis thaliana* Col-0 plants and the mutants *pad2*-*1* and *vtc2*-*1* throughout dark-induced senescence. H_2_O_2_ accumulation was first observed in cell walls (CW), plastids (*arrows*), the cytosol and along the tonoplast (*arrowheads*) 2 days and also 7 days after dark-induced senescence. *IS* intercellular spaces, *M* mitochondria, *N* nuclei, *P* plastids with or without starch (St), *V* vacuoles. *Bars* 1 μm

H_2_O_2_ distribution in leaf samples of *Arabidopsis* Col-0, and the mutant lines *pad2*-*1* and *vtc2*-*1* was visualized by CeCl_3_-staining. In *Arabidopsis* Col-0 and the mutant lines *pad2*-*1* and *vtc2*-*1* no dark precipitates caused by H_2_O_2_ accumulation could be found at the beginning of the experiment (Fig. 7). First accumulation of H_2_O_2_ could be detected 2 days after dark-induced senescence in the cell walls, plastids, cytosol, along the tonoplast and within vacuoles of *Arabidopsis* Col-0, *pad2*-*1* and *vtc2*-*1* (Fig. 7). A similar distribution but weaker staining was observed 7 days after dark-induced senescence (Fig. 7).

## Discussion

Overall, ascorbate- and glutathione-deficient mutants showed slightly more advanced signs of senescence when compared to wildtype plants. Both mutants showed earlier and stronger signs of yellowing, slightly lower pigment contents and plastid number after 4 and 7 days of dark-induced senescence. Additionally, *vtc2*-*1* mutants did not contain intact plastids at the end of the experiment. These results confirm the importance of antioxidants in regulating the process of senescence and are similar to what has been found in other plant species where senescence induced by pathogens, darkness or nutrient starvation was correlated with a decreased antioxidant capacity (del Río et al. 1998; Jiménez et al. 1998; Simova-Stoilova et al. 2005; Chen and Gallie 2006; del Río 2011; Großkinsky et al. 2012; Fotopoulos and Kanellis 2013; Simon et al. 2013; Ding et al. 2015). In this study the general decrease in antioxidative levels during dark-induced senescence was found to be correlated with H_2_O_2_ accumulation. The accumulation of H_2_O_2_ in leaves is a commonly found effect during dark-induced senescence (Fotopoulos and Kanellis 2013; Ding et al. 2015) and is responsible for the degeneration of lipids, chlorophyll, membranes and organelles and eventually cell death (Mach and Greenberg 2004; Zimmermann and Zentgraf 2005; Zentgraf 2007; Mhamdi et al. 2010). Further we could demonstrate that H_2_O_2_ initially (2 days after dark-induced senescence) accumulated in cell walls, plastids, the cytosol, the tonoplast, and within vacuoles, before symptoms were visible on the leaves. The diffusion and accumulation of H_2_O_2_ from plastids and peroxisomes into the cytosol and vacuoles was also observed during high light and drought stress (Heyneke et al. 2013; Koffler et al. 2014), and during advanced senescence induced by the infection of plants with *Botrytis cinerea* (Simon et al. 2013). As in all of these studies damages of the leaves such as necrosis and advanced yellowing could be observed, it seems that the diffusion of H_2_O_2_ into the cytosol contributes to cell death.

**Fig. 8:**
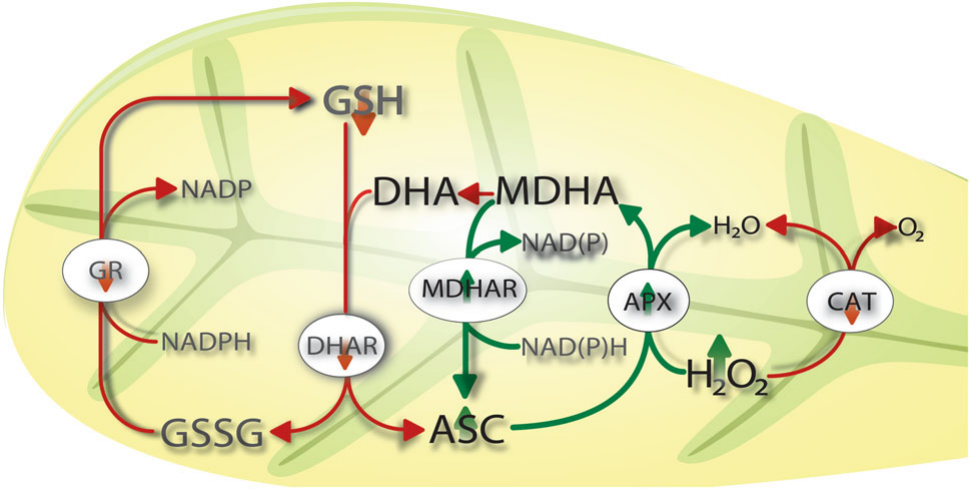
Changes in the ascorbate–glutathione cycle induced by dark-induced senescence. *Line* drawing proposing a model of changes in the ascorbate glutathione cycle during senescence. The strong decrease of glutathione contents within the first 24 h after dark-induced senescence followed by a strong decrease of the activity of GR, DHAR, and CAT leads to the accumulation of H_2_O_2_ which seems to be the main trigger for dark induced senescence. The increase of the activity of APX and MDHAR within the first 4 days after dark-induced senescence indicates that H_2_O_2_ accumulation during dark-induced senescence is controlled by APX through the oxidation of ascorbate (Asc) to MDHA and the subsequent reduction of MDHA to Asc by MDHAR

Senescence was more pronounced in the ascorbate-deficient mutant *vtc2*-*1* which, in contrast to the other plants, did not contain intact plastids or other cellular organelles at the end of the experiment. These results highlight the importance of ascorbate in controlling senescence. As glutathione levels and the activities of GR, DHAR, and CAT dropped in all plants throughout the experiment, increased APX activity became more important to counteract H_2_O_2_ accumulation induced by dark-induced senescence (Fig. 8). It has been demonstrated recently that reduced DHAR activity accelerates the onset of senescence and influences the rate of leaf aging through regulating the level of ROS (Chen and Gallie 2006). Similar effects have been observed in mutants with decreased GR contents which showed accelerated senescence correlated with the accumulation of H_2_O_2_ (Ding et al. 2015) The authors concluded that DHAR and GR play essential roles during leaf aging and senescence by controlling ROS levels in the tissue. Both mutants showed a much stronger increase in APX and MDHAR activity in comparison to the wildtype, indicating that the detoxification of H_2_O_2_ by APX through the oxidation of Asc to MDHA and the subsequent reduction of MDHA to Asc by MDHAR is the prominent pathway during dark induced senescence (Fig. 8). As this pathway is altered in the *vtc2*-*1* mutants by low levels of ascorbate the observed more pronounced signs of senescence in this mutant seem to be a logical consequence.

At the subcellular level it is remarkable that glutathione levels dropped significantly in all cell compartments within the first 24 h after dark treatment while ascorbate contents remained generally stable during the first few days of dark-induced senescence. These results indicate that a drop in glutathione contents plays an essential role in triggering and signaling senescence in the early stages. Especially, the breakdown of the antioxidative system in peroxisomes and mitochondria seems to be crucial for the induction of senescence. A drop of glutathione contents in these cell compartments was linked to an accumulation of ROS and to advanced senescence induced by darkness or pathogens (del Río et al. 1998; Jiménez et al. 1998; del Río 2011; Großkinsky et al. 2012; Simon et al. 2013). In this context it is interesting that glutathione contents in mitochondria of the *pad2*-*1* mutant decreased more slowly than in the other plants. Under control conditions this mutant develops a phenotype similar to the wildtype despite 90 % less glutathione contents in all cell compartments except mitochondria which show similar levels as the wildtype (Parisy et al. 2007; Zechmann et al. 2008). In contrast, it could be demonstrated that the *rml1* mutant which contains up to 97 % less glutathione in all cell compartments (including mitochondria) develops severe growth distortions (Vernoux et al. 2000; Zechmann and Müller 2010). Additionally, a strong accumulation of glutathione was observed in mitochondria of the *pad2*-*1* mutant during high light conditions and salt stress (Heyneke et al. 2013; Koffler et al. 2015). Thus, it appears that the accumulation and maintenance of high levels of glutathione in mitochondria of the *pad2*-*1* mutant form an important survival strategy of this mutant during environmental stress conditions. As glutathione degradation is carried out at the tonoplast, the plasma membrane, and within vacuoles (Ohkama-Ohtsu et al. 2007a, b; Ferretti et al. 2009; Destro et al. 2011; Tolin et al. 2013) the observed slower decline of glutathione levels in mitochondria of *pad2*-*1* indicate a distortion of glutathione transport from mitochondria into the cytosol.

Besides the depletion of glutathione in mitochondria and peroxisomes the strong drop of glutathione contents in nuclei during dark-induced senescence could also be an important trigger for senescence. The transition of cells from G1 to S phase was inhibited in cells treated with buthionine sulfoximine (BSO) which is an inhibitor of gamma-glutamylcysteine synthetase—a precursor for glutathione synthesis—and consequently lowers glutathione contents (Vernoux et al. 2000; Zechmann et al. 2006). Additionally, the inhibition of glutathione synthesis by BSO during G1 phase delayed cell division (Potters et al. 2004). Low glutathione levels were also found to disturb cell proliferation indicating that disturbances in redox balance in nuclei can lead to damages of DNA and possibly contribute to cell death that occurs during senescence (Diaz Vivancos et al. 2010). Thus, it is very likely that the strong decrease of glutathione observed in nuclei during dark-induced senescence contributes towards the senescence observed in this study.

All plants showed a strong reduction of plastid size and change in shape on semi- and ultra-thin sections throughout the experiment until they reached complete degradation. A reduction of plastid size and change in shape during developmental and induced senescence have also been observed in other plant species and seem to be a common sign of senescence (Inada et al. 1998a, b; Minamikawa et al. 2001; Wada et al. 2009). In addition, a strong change in chloroplast fine structure on ultra-thin sections could be observed with the TEM. Within the first few days of dark treatment, a reduction in size of starch grains could be observed on ultra-thin sections with the TEM in all plants which is a logical consequence of lack of photosynthesis under these conditions. A decrease in starch and chloroplast size was also observed during drought stress (Zellnig et al. 2004, 2010) which also negatively interferes with photosynthesis (Koffler et al. 2014). Consequently, a reduction of thylakoid membranes on ultra-thin sections and a reduction of pigment contents could be observed throughout the experiment starting within the first 2 days after dark treatment. The degradation of thylakoid membranes is accompanied by the downregulation of photosynthesis during senescence (Zimmermann and Zentgraf 2005) and related with the increase in plastoglobules, which could be observed with the TEM on ultra-thin cross sections of leaves of the investigated plants. An accumulation of numerous large plastoglobules was also observed during natural senescence and drought stress in different plant species (Pastori and del Río 1994; Inada et al. 1998a; Guiamét et al. 1999; Zellnig et al. 2004, 2010; Austin et al. 2006; Olmos et al. 2006) and their content was found to be altered during advanced senescence, since they displayed a diminished electron density (Fulgosi et al. 2012). An increase in plastoglobulus size was also observed recently in correlation with the exposure to high light conditions and decreasing thylakoid contents (Heyneke et al. 2013). Plastoglobules were shown to contain lipids derived from the degradation of thylakoids as well as chlorophyll, carotenoids, photosynthetic proteins (Guiamét et al. 1999), and proteins, involved in plant metabolism (Austin et al. 2006). Additionally, plastoglobules are considered to play an important role in the breakdown of carotenoids and oxidative stress defense (Ytterberg et al. 2006). They are also important for lipid biosynthesis and a storage compartment of thylakoid membranes during oxidative stress and senescence (Austin et al. 2006).

It can be concluded that dark-induced senescence is characterized by a general reduction in the antioxidative capacity in all cell compartments leading to an accumulation of ROS, especially in cell walls, plastids, and the cytosol. As glutathione levels and the activities of GR, DHAR, and CAT were strongly down-regulated throughout the experiment the most prominent pathway to detoxify H_2_O_2_ contents was controlled by APX through the oxidation of Asc to MDHA and the subsequent reduction of MDHA to Asc by MDHAR (Fig. 8). Additionally, *vtc2*-*1* mutants showed much stronger signs of senescence than the other plants. As ascorbate contents remained almost unchanged until the very end it seems that ascorbate rather than glutathione is the major agent regulating dark-induced senescence. As glutathione levels dropped rapidly within the first day of dark-induced senescence it seems that glutathione plays important roles in triggering and signaling senescence in the initial phase of dark-induced senescence. Considering evidence from other investigations the decline of glutathione in mitochondria, nuclei, plastids, and peroxisomes seems to be crucial in the development of senescence and should be dissected further.

### **Author contribution statement**

BZ and NL-E conceived of the study and participated in its design and coordination. NL-E carried out the electron and light microscopical work. BZ and NL-E performed quantitative and statistical analysis of the data and wrote the final manuscript.

## Electronic supplementary material

Below is the link to the electronic supplementary material.

**Fig. A1** **Semi-thin sections of leaves during dark induced senescence.** Representative light microscopic images of leaf sections from *Arabidopsis* Col-0 (first row) and the mutants *pad2*-*1* (second row) and *vtc2*-*1* (third row). Leaves at the beginning of the experiment are shown in the first column, leaves 4 d and 10 d after the beginning of dark induced senescence are shown in the second and third column, respectively. Bar = 50 μm. (JPEG 2696 kb)

**Fig. A2 TEM-micrographs showing changes in plastid fine structures during dark induced senescence.** Representative transmission electron micrographs of leaf sections from *Arabidopsis* Col-0 (first row), and the mutants *pad2*-*1* (second row) and *vtc2*-*1* (third row). Leaves at the beginning of the experiment (C = control) are shown in the first column, leaves 4 d, 7 d and 10 d after the beginning of dark induced senescence are shown in the second, third and fourth column, respectively. Plastids (P) show massive changes during the course of dark induced senescence such as decrease in starch (St), increase in number and size of plastoglobules (arrowheads), and their shape becomes roundish until their content is released into the vacuole (V) at the end of the experiment (arows). IS = intercellular space, M = mitochondria, N = nuclei. Bar = 1 μm (JPEG 2798 kb)

**Fig. A3 TEM-micrographs showing ascorbate labeling during dark induced senescence.** Representative transmission electron micrographs showing gold particles bound to ascorbate on leaf sections from the *Arabidopsis* Col-0 (first row), and the mutants *pad2*-*1* (second row) and *vtc2*-*1* (third row). Leaves at the beginning of the experiment (C = control) are shown in the first column, leaves 1 d, 7 d and 10 d after the beginning of dark induced senescence are shown in the second, third and fourth column, respectively. P = plastids with or without starch (St), M = mitochondria, N = nuclei, Px = peroxisomes, V = vacuoles. Bars = 1 µm (JPEG 2841 kb)

**Fig. A4 TEM-micrographs showing glutathione labeling during dark induced senescence.** Representative transmission electron micrographs showing gold particles bound to glutathione on leaf sections from the *Arabidopsis* Col-0 (first row), and the mutants *pad2*-*1* (second row) and *vtc2*-*1* (third row).). Leaves at the beginning of the experiment (C = control) are shown in the first column, leaves 1d, 7d and 10d after the beginning of dark induced senescence are shown in the second, third and fourth column, respectively. P = plastids with or without starch (St), M = mitochondria, N = nuclei, Px = peroxisomes, V = vacuoles. Bars = 1 µm (JPEG 2706 kb)

**Table A1: Analysis of significant differences between pigment contents, number of chloroplasts, and total areas of chloroplast fine structures during dark induced senescence.** Significant differences were calculated between wildtype plants, *pad2*-*1* and *vtc2*-*1* for samples within one sampling time point using the Mann–Whitney U-test. Samples which are significantly different from each other have no letters in common. *P* < 0.05 was regarded significant. Original data is shown in Fig. 2 and 3 (PDF 79 kb)

**Table A3: Analysis of significant differences between the activities of antioxidative enzymes during dark induced senescence.** Significant differences were calculated between wildtype plants, *pad2*-*1* and *vtc2*-*1* for samples within one sampling time point using the Mann–Whitney U-test. Samples which are significantly different from each other have no letters in common. *P* < 0.05 was regarded significant. Original data is shown in Fig. 6. CAT = catalase, GR = glutathione reductase, APX = ascorbate peroxidase, MDHAR = monodehydroascorbate reductase, and DHAR = dehydroascorbate reductase (PDF 80 kb)

**Table A2: Analysis of significant differences between subcellular ascorbate and glutathione contents during dark induced senescence.** Significant differences were calculated between wildtype plants, *pad2*-*1* and *vtc2*-*1* for samples within one sampling time point using the Mann–Whitney U-test. Samples which are significantly different from each other have no letters in common. *P* < 0.05 was regarded significant. Original data is shown in Fig. 4 and 5 (PDF 74 kb)

## Abbreviations

APX: Ascorbate peroxidase
Asc: Reduced ascorbate
BSO: Buthionine sulfoximine
CAT: Catalase
CeCl_3_: Cerium chloride
Chl: Chlorophyll
DHA: Dehydroascorbate
DHAR: Dehydroascorbate reductase
GR: Glutathione reductase
GSH: Reduced glutathione
GSSG: Glutathione disulfide
H_2_O_2_: Hydrogen peroxide
MDHA: Monodehydroascorbate
MDHAR: Monodehydroascorbate reductase
PCD: Programmed cell death
ROS: Reactive oxygen species
TEM: Transmission electron microscopy
