# Supplementary figures and images for: Compartment-specific investigations of antioxidants and hydrogen peroxide in leaves of Arabidopsis thaliana during dark-induced senescence
Source: Acta Physiol Plant. 2016 May 6;38:133. doi: 10.1007/s11738-016-2150-6 (PMC4859865; doi:10.1007/s11738-016-2150-6)

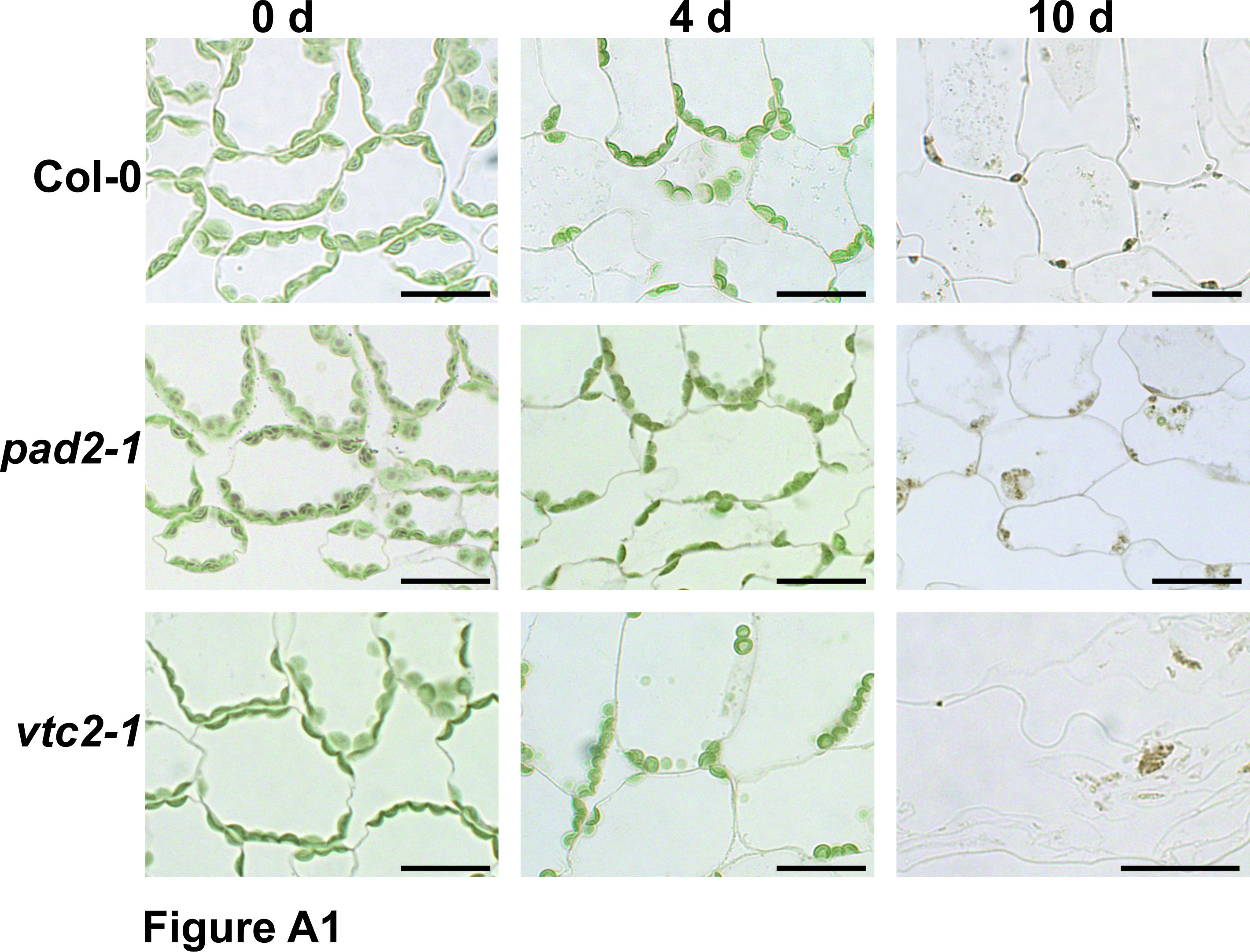

Supplement: Supplementary file 1 — Fig. A1 Semi-thin sections of leaves during dark induced senescence. Representative light microscopic images of leaf sections from Arabidopsis Col-0 (first row) and the mutants pad2-1 (second row) and vtc2-1 (third row). Leaves at the beginning of the experiment are shown in the first column, leaves 4 d and 10 d after the beginning of dark induced senescence are shown in the second and third column, respectively. Bar = 50 μm. (JPEG 2696 kb) [file 11738_2016_2150_MOESM1_ESM.jpg]

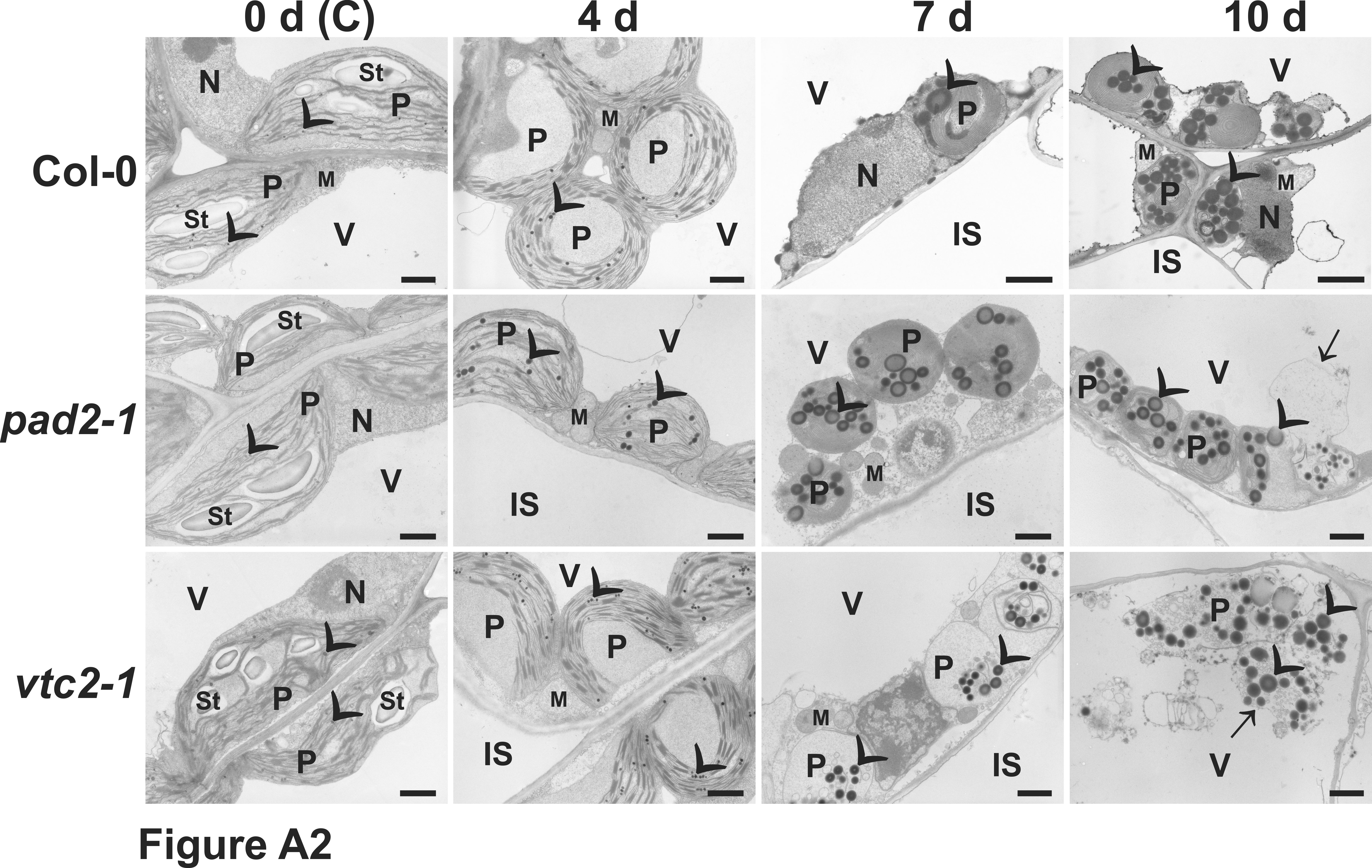

Supplement: Supplementary file 2 — Fig. A2 TEM-micrographs showing changes in plastid fine structures during dark induced senescence. Representative transmission electron micrographs of leaf sections from Arabidopsis Col-0 (first row), and the mutants pad2-1 (second row) and vtc2-1 (third row). Leaves at the beginning of the experiment (C = control) are shown in the first column, leaves 4 d, 7 d and 10 d after the beginning of dark induced senescence are shown in the second, third and fourth column, respectively. Plastids (P) show massive changes during the course of dark induced senescence such as decrease in starch (St), increase in number and size of plastoglobules (arrowheads), and their shape becomes roundish until their content is released into the vacuole (V) at the end of the experiment (arows). IS = intercellular space, M = mitochondria, N = nuclei. Bar = 1 μm (JPEG 2798 kb) [file 11738_2016_2150_MOESM2_ESM.jpg]

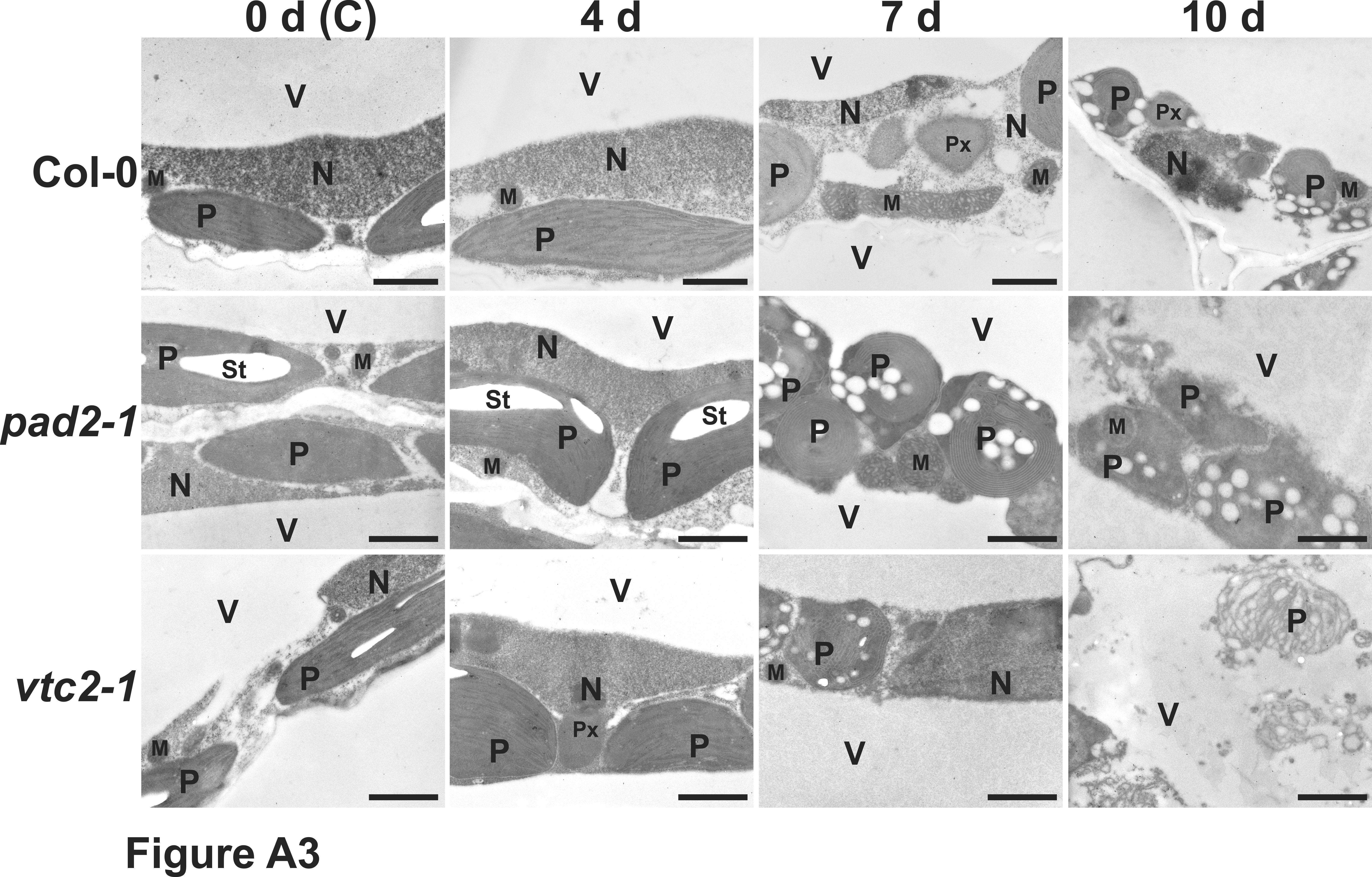

Supplement: Supplementary file 3 — Fig. A3 TEM-micrographs showing ascorbate labeling during dark induced senescence. Representative transmission electron micrographs showing gold particles bound to ascorbate on leaf sections from the Arabidopsis Col-0 (first row), and the mutants pad2-1 (second row) and vtc2-1 (third row). Leaves at the beginning of the experiment (C = control) are shown in the first column, leaves 1 d, 7 d and 10 d after the beginning of dark induced senescence are shown in the second, third and fourth column, respectively. P = plastids with or without starch (St), M = mitochondria, N = nuclei, Px = peroxisomes, V = vacuoles. Bars = 1 µm (JPEG 2841 kb) [file 11738_2016_2150_MOESM3_ESM.jpg]

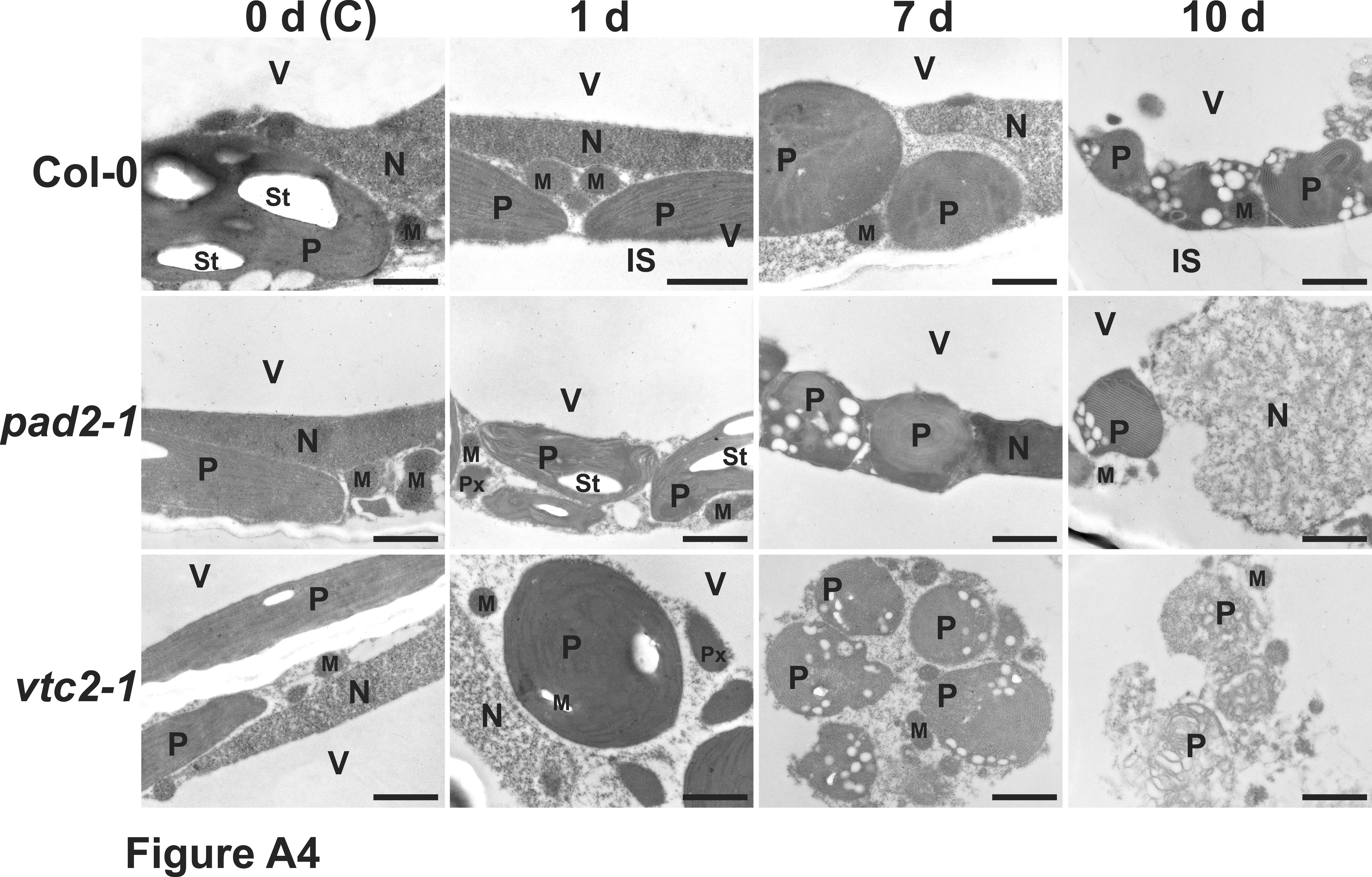

Supplement: Supplementary file 4 — Fig. A4 TEM-micrographs showing glutathione labeling during dark induced senescence. Representative transmission electron micrographs showing gold particles bound to glutathione on leaf sections from the Arabidopsis Col-0 (first row), and the mutants pad2-1 (second row) and vtc2-1 (third row).). Leaves at the beginning of the experiment (C = control) are shown in the first column, leaves 1d, 7d and 10d after the beginning of dark induced senescence are shown in the second, third and fourth column, respectively. P = plastids with or without starch (St), M = mitochondria, N = nuclei, Px = peroxisomes, V = vacuoles. Bars = 1 µm (JPEG 2706 kb) [file 11738_2016_2150_MOESM4_ESM.jpg]
