# Supplementary material for: Compartment-specific investigations of antioxidants and hydrogen peroxide in leaves of Arabidopsis thaliana during dark-induced senescence
Source: Acta Physiol Plant. 2016 May 6;38:133. doi: 10.1007/s11738-016-2150-6 (PMC4859865; doi:10.1007/s11738-016-2150-6)
Supplement: Supplementary file 5 — Table A1: Analysis of significant differences between pigment contents, number of chloroplasts, and total areas of chloroplast fine structures during dark induced senescence. Significant differences were calculated between wildtype plants, pad2-1 and vtc2-1 for samples within one sampling time point using the Mann–Whitney U-test. Samples which are significantly different from each other have no letters in common. P < 0.05 was regarded significant. Original data is shown in Fig. 2 and 3 (PDF 79 kb) [file 11738_2016_2150_MOESM5_ESM.pdf]

mg g^{-1} FW	Chlorophyll a			Chlorophyll b			Days in darkness
	Col-0	*pad2-1*	*vtc2-1*	Col-0	*pad2-1*	*vtc2-1*	
0 d	a	a	a	a	b	b	0 d
1 d	ab	b	a	ab	b	a	1 d
2 d	a	b	a	a	b	a	2 d
4 d	a	b	a	a	c	b	4 d
7 d	b	a	b	a	b	b	7 d
10 d	a	a	a	ab	b	a	10 d
mg g^{-1} FW	Carotenoids			Total chlorophyll			Days in darkness
	Col-0	*pad2-1*	*vtc2-1*	Col-0	*pad2-1*	*vtc2-1*	
0 d	a	b	b	a	b	b	0 d
1 d	ab	b	a	a	a	b	1 d
2 d	a	b	a	a	a	b	2 d
4 d	ab	c	b	a	b	ab	4 d
7 d	a	b	b	a	b	b	7 d
10 d	a	a	a	a	a	a	10 d
Number of chloroplasts	Spongy parenchyma			Palisade parenchyma			Days in darkness
	Col-0	*pad2-1*	*vtc2-1*	Col-0	*pad2-1*	*vtc2-1*	
0 d	a	b	c	b	a	a	0 d
1 d	b	a	b	a	a	a	1 d
2 d	a	a	b	a	b	c	2 d
4 d	a	a	a	a	a	a	4 d
7 d	a	b	c	a	b	c	7 d
10 d	a	a	-	a	a	-	10 d
Total of area in μm^2	Plastids			Thylakoids			Days in darkness
	Col-0	*pad2-1*	*vtc2-1*	Col-0	*pad2-1*	*vtc2-1*	
0 d	a	b	b	a	b	b	0 d
1 d	a	ab	b	a	b	b	1 d
2 d	a	a	a	a	b	a	2 d
4 d	a	c	b	a	b	b	4 d
7 d	a	b	a	a	b	b	7 d
10 d	a	b	-	a	a	-	10 d
Total of area in μm^2	Starch			Plastoglobules			Days in darkness
	Col-0	*pad2-1*	*vtc2-1*	Col-0	*pad2-1*	*vtc2-1*	
0 d	a	a	b	a	a	a	0 d
1 d	b	a	b	a	a	a	1 d
2 d	b	a	b	a	a	a	2 d
4 d	a	a	a	a	a	a	4 d
7 d	a	a	a	b	a	b	7 d
10 d	a	a	-	a	a	-	10 d

Table A1
