# Supplementary material for: Compartment-specific investigations of antioxidants and hydrogen peroxide in leaves of Arabidopsis thaliana during dark-induced senescence
Source: Acta Physiol Plant. 2016 May 6;38:133. doi: 10.1007/s11738-016-2150-6 (PMC4859865; doi:10.1007/s11738-016-2150-6)
Supplement: Supplementary file 6 — Table A3: Analysis of significant differences between the activities of antioxidative enzymes during dark induced senescence. Significant differences were calculated between wildtype plants, pad2-1 and vtc2-1 for samples within one sampling time point using the Mann–Whitney U-test. Samples which are significantly different from each other have no letters in common. P < 0.05 was regarded significant. Original data is shown in Fig. 6. CAT = catalase, GR = glutathione reductase, APX = ascorbate peroxidase, MDHAR = monodehydroascorbate reductase, and DHAR = dehydroascorbate reductase (PDF 80 kb) [file 11738_2016_2150_MOESM6_ESM.pdf]

Mitochondria	Glutathione Col-0	Glutathione *pad2-1*	Glutathione *vtc2-1*	Ascorbate Col-0	Ascorbate *pad2-1*	Ascorbate *vtc2-1*	Days in darkness
0 d	a	a	b	a	a	b	0 d
1 d	b	a	b	a	a	a	1 d
2 d	b	a	b	a	b	b	2 d
4 d	b	a	b	a	a	a	4 d
7 d	a	b	b	a	b	b	7 d
10 d	a	b	-	a	b	-	10 d

Plastids	Glutathione Col-0	Glutathione *pad2-1*	Glutathione *vtc2-1*	Ascorbate Col-0	Ascorbate *pad2-1*	Ascorbate *vtc2-1*	Days in darkness
0 d	b	c	a	a	a	b	0 d
1 d	a	a	b	a	a	a	1 d
2 d	b	a	b	a	a	a	2 d
4 d	b	a	b	a	a	b	4 d
7 d	a	a	b	a	a	b	7 d
10 d	a	b	-	a	b	-	10 d

Nuclei	Glutathione Col-0	Glutathione *pad2-1*	Glutathione *vtc2-1*	Ascorbate Col-0	Ascorbate *pad2-1*	Ascorbate *vtc2-1*	Days in darkness
0 d	a	b	a	a	a	b	0 d
1 d	a	b	b	a	a	c	1 d
2 d	a	a	a	a	a	c	2 d
4 d	a	a	a	a	a	b	4 d
7 d	a	a	b	a	a	b	7 d
10 d	a	b	-	b	b	-	10 d

Peroxisomes	Glutathione Col-0	Glutathione *pad2-1*	Glutathione *vtc2-1*	Ascorbate Col-0	Ascorbate *pad2-1*	Ascorbate *vtc2-1*	Days in darkness
0 d	b	c	a	a	a	b	0 d
1 d	a	b	b	a	b	c	1 d
2 d	ab	b	a	a	a	a	2 d
4 d	a	a	a	a	b	b	4 d
7 d	a	a	a	a	ab	b	7 d
10 d	a	a	-	a	b	-	10 d

Cytosol	Glutathione Col-0	Glutathione *pad2-1*	Glutathione *vtc2-1*	Ascorbate Col-0	Ascorbate *pad2-1*	Ascorbate *vtc2-1*	Days in darkness
0 d	a	c	b	a	a	b	0 d
1 d	a	b	b	b	a	c	1 d
2 d	a	b	a	a	b	c	2 d
4 d	a	b	c	a	b	b	4 d
7 d	a	a	a	a	b	b	7 d
10 d	a	a	-	a	b	-	10 d

Vacuoles	Ascorbate Col-0	Ascorbate *pad2-1*	Ascorbate *vtc2-1*	Days in darkness
0 d	a	a	a	0 d
1 d	b	a	b	1 d
2 d	a	a	a	2 d
4 d	a	a	a	4 d
7 d	a	b	b	7 d
10 d	a	b	-	10 d

Table A2
